# Liver-Kidney Transplantation in Primary Hyperoxaluria Type-1: Case Report and Literature Review

**Published:** 2011-08-01

**Authors:** D. Siegal, W. S. Su, D. DaBreo, M. Puglia, L. Gregor, A. S. Gangji

**Affiliations:** 1 *Division of Nephrology and Transplantation, McMaster University and St. Joseph’s Health Care, Hamilton, Ontario, Canada*; 2 *Division of Nephrology, Grand River Hospital, Kitchener, Ontario, Canada*; 3 *Division of Gastroenterology, McMaster University, Ontario Canada*

**Keywords:** Primary hyperoxaluria, renal transplantation, liver transplantation

## Abstract

Primary hyperoxaluria type-1 (PH1) is a rare inherited autosomal recessive disorder in which a deficiency of the hepatic enzyme alanine-glyoxylate aminotransferase leads to endogenous oxalate overproduction, renal failure, systemic oxalate deposition and death. As hemodialysis provides insufficient oxalate clearance, patients ultimately require both liver and kidney transplantation for correction of the metabolic abnormality and oxalate excretion. Herein, we describe a young adult male with end-stage renal disease and systemic oxalosis causing progressive disabling multi-organ dysfunction while awaiting transplantation. We review the literature regarding liver-kidney transplantation and suggest that for patients with PH1, a standardized assessment of organ dysfunction and functional impairment may improve identification of patients requiring urgent transplantation thereby reducing the morbidity and mortality that can occur with delayed transplantation.

## Introduction

**Figure 1:**
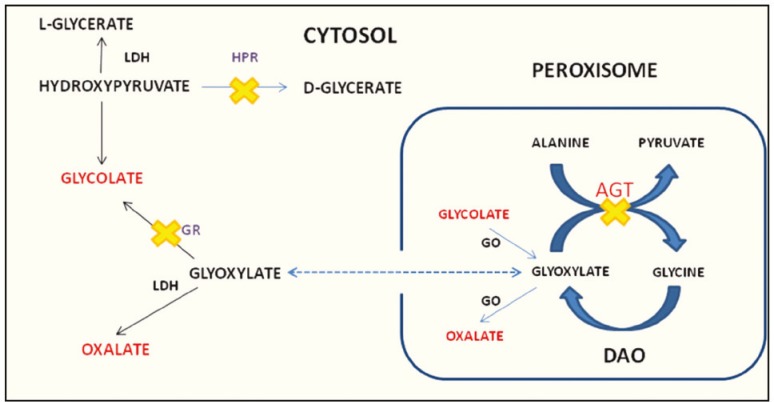
Glyoxylate metabolism in the hepatic peroxisome. In PH1, deficiency or mistargeting of AGT results in decreased transamination of glyoxylate to glycine, and increased production of oxalate. AGT: Alanine-glyoxylate aminotransferase; DAO: D-amino oxidase; HPR: Hydroxyl pyruvate reductase; GO: Glycolate oxidase; GR: Glyoxylate reductase

Primary hyperoxaluria type-1 (PH1) is a rare autosomal recessive inherited metabolic disorder occurring in 0.11 to 0.26 per 100,000 births [1]. It results from deficiency or mistargeting of the hepatic peroxisomal enzyme alanine-glyoxylate aminotransferase (AGT) which normally catalyzes the transformation of glyoxylate to glycine [2]. Deficiency or mistargeting of this enzyme leads to reduced transamination of glyoxylate to glycine, and increased production of the insoluble by-product oxalate (Fig 1). Oxalate is excreted exclusively by the kidneys and endogenous overproduction leads to supersaturation of urine with oxalate and subsequent oxalate urolithiasis, nephrocalcinosis, renal tubular damage, renal failure and death [2]. When glomerular filtration rate (GFR) is less than 25 mL/min/1.73 m^2^, production of oxalate far exceeds renal oxalate clearance and a rapid decline in renal function ensues [3]. End-stage renal disease (ESRD) is accompanied by systemic calcium oxalate deposition in extra-renal tissues including the skin, bone marrow, myocardium, nervous system, skeletal muscle, blood vessels and retina [4]. Patients lacking AGT activity require both liver and kidney transplantation to correct the metabolic abnormality and allow for oxalate elimination. The goal of this case presentation is to discuss the importance of identifying patients with ESRD secondary to PH1 who may benefit from urgent liver-kidney transplantation. Current pre-transplantation assessment methods such as the Model for End-Stage Liver Disease (MELD) are based on the severity of liver failure score and do not incorporate the extent of systemic disease associated with PH1 [5]. Therefore, we suggest the use of an alternative standardized assessment of multi-organ dysfunction and functional status to prioritize patients in order to decrease the interval to transplantation and associated morbidity and mortality.

## CASE REPORT

**Figure 2:**
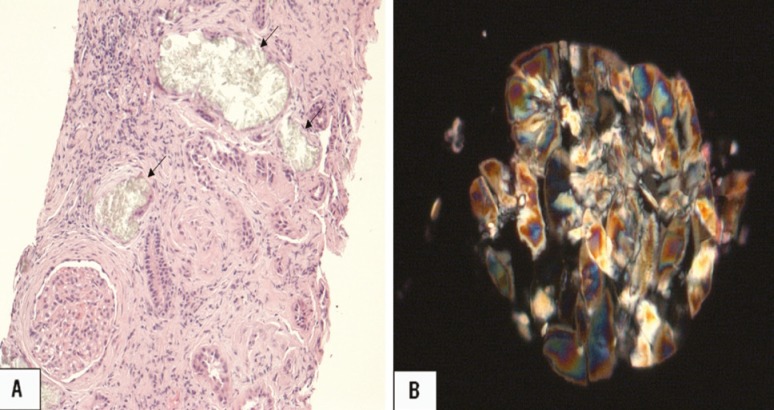
Renal biopsy specimen demonstrating oxalate deposition (arrows) visualized under A) light microscopy and B) polarized light

An 18-year-old East Indian male was referred for consideration of renal transplantation. He presented with a 2-week history of vomiting and malaise, and a serum creatinine of 42.8 mg/dL requiring urgent hemodialysis. A renal biopsy demonstrated severe tubulo-interstital disease with extensive oxalate crystal deposition in the renal tubules (Fig 2). He progressed rapidly to anuria and was unable to complete a 24-hour urine collection. The skin and retinal biopsies confirmed significant extra-renal oxalate deposition.

A diagnosis of PH1 was suspected and investigations revealed an elevated plasma oxalate level of 81.9 (normal <1.8) μmol/L. Confirmation of the diagnosis was established by liver biopsy demonstrating decreased AGT activity (2.7 µmol/hr/mg protein; normal 19.1–47.9 μmol/hr/mg protein) and negative AGT immunoreactivity (Department of Clinical Biochemistry, University College London Hospitals NHS Foundation Trust). Genotyping revealed that the patient was homozygous for the c.302T>C (Leu101Pro) mutation in exon 2 of the *AGXT* gene by sequence analysis. Both parents were heterozygous for the mutation. 

**Figure 3:**
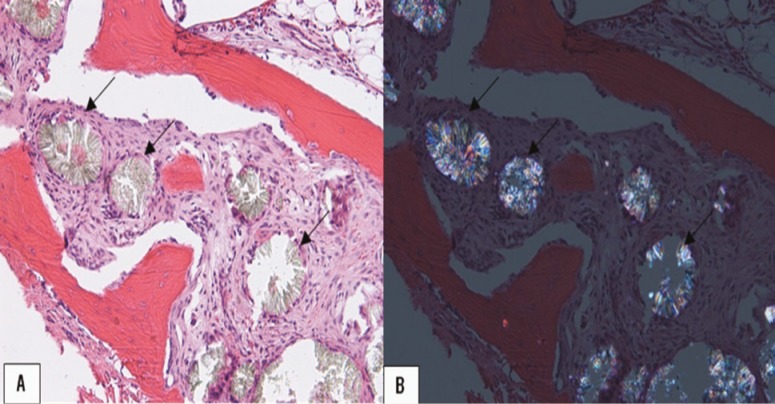
Bone marrow biopsy specimen demonstrating oxalate deposition (arrows) visualized under A) light microscopy and B) polarized light

An urgent referral was made for liver-kidney transplantation and the patient was placed on the waiting list for a deceased donor liver transplant, but did not meet criteria for priority listing. In the interim, daily hemodialysis was initiated in an attempt to increase oxalate clearance. Over the next 20 months, the patient’s functional status deteriorated significantly due to myopathy as confirmed by electromyography; he developed a restrictive ventilatory disorder based on pulmonary function testing, and erythropoietin-resistant anemia secondary to oxalate deposition as demonstrated on bone marrow biopsy (Fig 3). Echocardiography showed severe left ventricular hypertrophy and myocardial texture suspicious for cardiac oxalosis which was a marked interval change over the previous 12 months. 

Given the significant progression of multi-organ involvement over a short period, a request was made for consideration of urgent transplantation. The patient did not meet the priority liver transplant listing criteria, but an exception was made due to the extent of multi-organ disease involvement. Approximately 3.5 years after dialysis initiation, the patient received a simultaneous liver-kidney transplant. The post-operative course was complicated by delayed renal allograft function requiring dialysis for five weeks post-transplantation. A renal transplant biopsy demonstrated interstitial tubular calcium oxalate deposition consistent with recurrent disease. Renal function improved gradually with a serum creatinine level of 1.91 mg/dL two months post-transplantation.

## DISCUSSION

PH1 is a rare cause of ESRD. Symptoms of PH1 are typically secondary to urolithiasis (renal colic, hematuria, urinary tract infection, acute renal failure from complete obstruction) [2]. Patients can also present with ESRD (up to one-third of patients in some case series) with a median age of 25 to 40 years [6]. Because the efficacy of treatment is dependent upon early diagnosis, a high degree of suspicion must be maintained with a history of recurrent nephrolithiasis, radiological evidence of nephrocalcinosis, or ESRD with a history of renal stones or calcinosis [2]. According to US Renal Data System (USRDS) data, projected survival of PH1 patients without transplantation is 40% at five years and 20% at nine years after diagnosis of ESRD [7]. 

Patients with PH1 have markedly increased urinary oxalate excretion of greater than 1 mmol/1.73 m^2^ per day with some patients excreting as much as 1.5 to 3 mmol/1.73 m^2^ per day (normal oxalate excretion is <0.5 mmol/1.73 m^2^ per day) [8]. However, renal insufficiency is associated with a progressive decline in urinary oxalate excretion which limits its diagnostic efficacy. Moreover, patients presenting with ESRD may be anuric. In this setting, increased plasma oxalate concentrations support, but do not confirm the diagnosis of PH1 as renal insufficiency alone can result in increased plasma oxalate levels. 

Until recently, liver biopsy and *in vitro* measurement of AGT enzymatic activity provided a definitive diagnosis of PH1. However, molecular genetic testing is increasingly utilized for the diagnosis. At least 146 mutations associated with PH1 have now been described [9]. As the *AGXT* gene is relatively small (approximately 10 kb), whole-gene sequence analysis and targeted mutation analysis are readily-available options to detect both common and rare mutations [9]. 

Accumulated body oxalate is the major determinant of long-term complications in patients with PH1 [10]. In patients who develop ESRD, removal of oxalate by standard maintenance hemodialysis (950–1400 mmol/day) is insufficient to compensate for the excessive oxalate burden (3,500–7,500 mmol/day) and systemic oxalosis occurs with deposition in extra-renal organs including the skin, blood vessels, bone, bone marrow, retina, myocardium, and skeletal muscle [3]. Manifestations of systemic oxalosis including livedoreticularis, digital infarcts, osteopathy, pancytopenia, blindness, cardiomyopathy, cardiac valvular dysfunction and heart block are important causes of morbidity, and cardiovascular disorders represent the most common cause of death [11]. Oxalate clearance is greater with hemodialysis than with peritoneal dialysis, but these modalities neither compensate for excessive endogenous production, nor reduce total body oxalate stores [12]. 

Isolated kidney transplantation increases renal clearance of oxalate, but is limited by disease recurrence and allograft loss due to ongoing excess urinary oxalate excretion. Data from the European Dialysis and Transplant Association demonstrated poor outcomes of isolated kidney transplantation (mostly deceased donor renal transplants) in Europe in the 1980s with 3-year patient and graft survival of 74% and 20%, respectively [11]. Failure was primarily due to rejection (33%) and recurrence of the primary renal disease (31%). In the US, USRDS data showed that 6-year survival was 84% for patients and 51% for grafts, while 10-year graft survival was only 35% [7]. According to data from the United Network for Organ Sharing (UNOS) database (1992-2005), patients with oxalosis had decreased 5-year graft survival (52.1%) compared to all kidney transplant recipients (78.7%) [2]. However, recent data from the International Primary Hyperoxaluria Registry (IPHR) showed an increased proportion of functioning isolated kidney grafts at three years post-transplantation in transplants conducted since 2000 (84% after 2000, 55% before 2000), which may reflect changes in immunosuppressive therapy and/or earlier detection of PH1 prior to transplantation [13].

Combined liver-kidney transplantation is the sole treatment option available to correct the metabolic abnormality, provide renal replacement therapy, and allow for normalization of oxalate excretion in patients with PH1. Combined liver-kidney transplantation provides improved graft survival compared to kidney transplantation alone [13-17]. USRDS registry data report a significantly better 8-year death-censored graft survival rate of 76% following liver-kidney transplantation compared to 47.9% following isolated kidney transplantation [16]. European studies reported overall patient survival rates of 80% at five years and 69% at 10 years following liver-kidney transplantation [10,14,18]. Graft survival rates at 5 and 10 years post-transplantation were 72% and 60%, respectively [10,14]. Recent IPHR data from 1976 to 2009 also demonstrate better death-censored 3-year graft survival with liver-kidney (95%) *vs*. isolated kidney (56%) transplantation [13]. Among combined liver-kidney transplants, more recent transplants (2000 to 2009) were functioning at three years compared to earlier transplants (before 2000) (84% *vs.* 55%). In this report, however, three patients in the liver-kidney transplant group died with functioning grafts resulting in lower long-term (7-year) patient survival in the liver-kidney (67%) *vs*. isolated kidney transplant patients (88%).

Liver-kidney transplantation may be performed simultaneously (concurrent) or sequentially (liver then kidney). There is limited data regarding the superiority of either approach. Benefits of the simultaneous strategy include immediate cure of the metabolic defect and renal replacement. A functional renal graft provided by the simultaneous approach may reduce the morbidity and mortality associated with the presence of renal impairment after liver transplantation. However, mobilization of tissue oxalate and continued hyperoxaluria for months or years post-transplant can lead to renal allograft damage [2,10]. One series of pediatrics simultaneous liver-kidney transplants demonstrated decreased renal function at 3, 6 and 12 months post-transplantation in patients with PH1 compared to those with other diagnoses [19]. 

Sequential liver-kidney transplantation may be advantageous in certain clinical scenarios including patients with ESRD and a large total body oxalate burden, such as the patient in our case. Using this strategy, liver transplantation may allow clearing of tissue oxalate and protection of the subsequent renal allograft from the effects of hyperoxaluria. Despite the theoretical benefits of this approach, there is limited data in support of this strategy. UNOS registry data of all simultaneous and sequential liver-kidney transplantation between 1996 and 2003 demonstrated decreased mean±SD kidney half-life (6.6±0.9 *vs*. 11.7±1.3 years) and increased incidence of chronic rejection (4.6% *vs.* 1.6%) in sequential *vs*. simultaneous transplants [20]. One- and 3-year death censored renal graft survival was not different between the sequential (92% at 1-year, 86% at 3-year) and simultaneous (91% at 1-year and 86% at 3-year) groups. However, this data was not limited to PH1 patients. 

Pre-emptive liver transplantation has been suggested as an approach to avoid renal damage and oxalate deposition prior to the onset of significant renal impairment. However, donor scarcity and delays in transplantation ultimately lead to the onset of significant renal dysfunction prior to organ availability. Living-related organ donation is another strategy which may maximize potential benefit to the recipient and decrease the interval to transplantation. For both strategies, the replacement of a functional liver to correct a metabolic abnormality raises important ethical concerns including peri-operative risk and the need for long-term immunosuppression. PH1 has an unpredictable clinical course even when the genotype is known and the morbidity and mortality risks must be weighed against the possibility that a patient might have lived for years without transplantation and the risks of long-term immunosuppression. 

As demonstrated by the present case, significant morbidity can occur rapidly in patients awaiting transplantation due to ongoing systemic oxalate deposition. Our patient developed cardiomyopathy and skeletal myopathy within only one year of awaiting transplantation on hemodialysis. As systemic oxalate deposition is the major determinant of long-term outcome, prompt referral for liver-kidney transplantation is essential. Data from the European PH1 Transplant Registry demonstrates that prolonged duration of dialysis prior to transplantation is associated with worse clinical status at the time of transplantation and decreased survival post-transplant [10].

According to the American Association for the Study of Liver Disease (AASLD) practice guidelines, liver transplantation is indicated for any cause of acute or chronic liver failure [5]. Despite the presence of normal liver function, hyperoxaluria is included as an indication for liver transplantation. The need for liver transplantation is assessed using clinical scoring systems including the prognostic MELD score which incorporates serum creatinine, serum bilirubin and international normalized ratio for prothrombin time [5]. Using this model, patients are assigned a score from 6 to 40 associated with 3-month survival rates of 90% to 7%, respectively. Because PH1 patients do not have liver disease *per se*, their calculated MELD score does not reflect the multi-system organ failure that occurs with systemic oxalosis, nor the true urgency of transplantation. In 2006, standardized MELD score exceptions were proposed for patients with PH1 meeting specific criteria including AGT deficiency determined by liver biopsy [21]. Briefly, patients older than one year of age and fulfilling the criteria will receive initial MELD scores equivalent to a 10% mortality risk (no ESRD) or 15% mortality risk (ESRD) with 10% mortality equivalents added every three months. While the MELD exceptions may expedite priority listing of PH1 patients for liver and liver-kidney transplantation, they do not incorporate the multi-system organ dysfunction occurring with systemic oxalosis. Importantly, patients with ESRD present with variable degrees of systemic oxalate deposition and the extent of end-organ dysfunction in individual patients is an important consideration for urgency of transplantation. Studies are warranted to determine whether the mortality equivalents of the MELD exceptions correspond well with patient survival. 

Given the disparity between the clinical features of PH1 and the model used to assign priority for liver transplantation, consideration should be given to the use of a separate scoring system to assess urgency of transplantation based on the extent of multi-organ dysfunction and functional impairment. For example, the Index of Coexistent Disease (ICED), assesses the severity of patient comorbidity and functional impairment, and has been shown to predict survival in hemodialysis patients [22,23]. While the ICED has not been validated specifically for patients with ESRD secondary to PH1, it is advantageous in that it includes measures of both disease severity and physical function, is based on readily available clinical information and does not require training for administration. Utilization of a standardized assessment tool such as the ICED would provide information regarding disease burden, overall quality of life and survival, which may help stratify patients in need of transplantation. Moreover, it may help direct scarce organ allocation to patients with the greatest need.
